# Supplementary material for: Development and Reproduction of a Japanese Strain of Ctenolepisma calvum (Ritter, 1910) at Room Temperature
Source: Insects. 2023 Jun 16;14(6):563. doi: 10.3390/insects14060563 (PMC10299600; doi:10.3390/insects14060563)
Supplement: Supplementary file 1 [file insects-14-00563-s001.zip › Figure S1_230510.pdf]

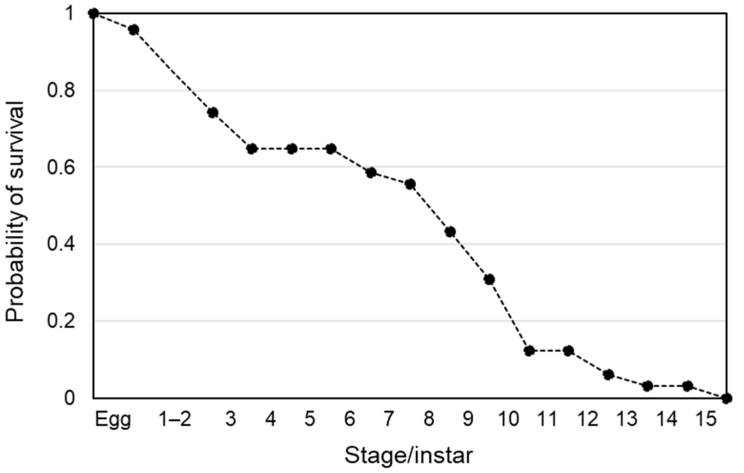

Figure S1. Probability of survival from eggs calculated based on the data in Tables 1 and 3 (also refer to Tables S5 for sample size). Note that these data were obtained from separate experiments and combined to estimate the probability of survival after hatching.
