# Supplementary material for: Development and Reproduction of a Japanese Strain of Ctenolepisma calvum (Ritter, 1910) at Room Temperature
Source: Insects. 2023 Jun 16;14(6):563. doi: 10.3390/insects14060563 (PMC10299600; doi:10.3390/insects14060563)
Supplement: Supplementary file 1 [file insects-14-00563-s001.zip › Table S1_230510.pdf]

Table S1. Egg period and survival of eggs and young (1st–2nd instar) juveniles

Date of oviposition	Number of eggs laid	Egg period (days)	Number of hatched eggs	Average temperature in egg period (°C)	Number of individuals that survived to 3rd instar per number of 1st instar individuals
Jul. 17, 2019	5	58	3	25.4	3/4
		unknown	1	-	
Oct. 7, 2019	7	73	7	23.9	Not measured
Oct. 11, 2019	1	73	1	23.7	Not measured
Nov. 17, 2019	18	64	16	22.0	Not measured
		71	2	22.0	
Apr. 2, 2020	11	85	6	23.1	8/9*
		87	4	23.1	
May 14, 2020	10	57	5	24.9	6/9
		59	4	24.9	
Jun. 8, 2020	9	56	1	25.1	5/9
		57	3	25.2	
		58	5	25.2	
Jun. 19, 2020	9	54	3	25.3	9/9
		55	3	25.3	
		56	3	25.3	

*One of the 10 individuals died by mishandling.
