# Supplementary material for: Development and Reproduction of a Japanese Strain of Ctenolepisma calvum (Ritter, 1910) at Room Temperature
Source: Insects. 2023 Jun 16;14(6):563. doi: 10.3390/insects14060563 (PMC10299600; doi:10.3390/insects14060563)
Supplement: Supplementary file 1 [file insects-14-00563-s001.zip › Table S2_230510.pdf]

Table S2. Time of ecdysis and average temperature during instar periods in each individual

Individual number	Date of ecdysis to the following instars (upper row) and average temperature (°C) in the instar periods (lower row)														
	1st instar (hatching)	2nd instar	3rd instar	4th instar	5th instar	6th instar	7th instar	8th instar	9th instar	10th instar	11th instar	12th instar	13th instar	14th instar	15th instar
	-	-	-	Jul 22, 2019	Aug 20, 2019	Sep 6, 2019	Sep 25, 2019	Oct 16, 2019	Nov 18, 2019	Mar 16, 2020	May 23, 2020	Jun 29, 2020			
#1												(Jul 21 d)			
	-	-	-	25.6	25.1	25.5	25.2	24.5	21.8	21.8	25.1				
	-	-	-	Jul 23, 2019	Aug 20, 2019	Sep 6, 2019	Sep 26, 2019	Oct 21, 2019	Nov 25, 2019	Mar 18, 2020	May 27, 2020	Jul 1, 2020			
#2												(Aug 12 d)			
	-	-	-	25.6	25.1	25.5	25.1	24.3	21.6	22.0	25.1				
	-	-	-	Jul 22, 2019	Aug 30, 2019	Sep 25, 2019	Oct 15, 2019	Nov 7, 2019	Dec 16, 2019	Mar 5, 2020	May 20, 2020	Jun 29, 2020	Aug 12, 2020	Oct 27, 2020	Mar 27, 2021
#3															(Apr 24 d)
	-	-	-	25.5	25.4	25.2	24.7	23.2	21.5	21.7	24.9	25.3	25.6	21.9	
	-	-	-	Jul 22, 2019	Sep 25, 2019	Oct 15, 2019	Nov 4, 2019	Dec 3, 2019	Mar 2, 2020	Apr 26, 2020	May 27, 2020	Jul 13, 2020	Sep 22, 2020		
#4													(Mar 8 d)		
	-	-	-	25.5	25.2	24.7	23.7	21.6	20.9	23.5	25.1	25.8			
	-	-	-	Sep 12, 2019	Oct 4, 2019	Oct 30, 2019	Nov 25, 2019	Jan 10, 2020	Mar 10, 2020	May 19, 2020					
#5										(Jun 5 d)					
	-	-	-	25.4	24.9	24.1	21.9	21.5	21.7						
	-	-	-	Sep 25, 2019	Oct 16, 2019	Nov 7, 2019	Dec 5, 2019	Jan 22, 2020	Mar 19, 2020	May 20, 2020					
#6										(Jun 2 d)					
	-	-	-	25.2	24.7	23.6	21.5	21.5	21.8						
	-	-	Aug 27, 2019	Sep 30, 2019	Oct 25, 2019	Nov 14, 2019	Dec 16, 2019	Feb 7, 2020	Mar 24, 2020	May 18, 2020					
#7										(Jun 16 d)					
	-	-	25.4	25.0	24.5	23.0	21.4	21.5	21.7						
	Sep 13, 2019	Sep 18, 2019	Sep 30, 2019	Oct 21, 2019	Nov 7, 2019	Dec 10, 2019									
#8						(Feb 5 d)									
	25.6	25.4	25.0	24.7	23.4										
	Sep 13, 2019	Sep 18, 2019	Sep 30, 2019	Oct 21, 2019	Nov 7, 2019	Dec 16, 2019	Mar 6, 2020	May 14, 2020	Jun 12, 2020						
#9									(Jun 22 d)						
	25.6	25.4	25.0	24.7	23.2	21.4	21.4	24.8							
	Sep 13, 2019	Sep 18, 2019	Sep 30, 2019	Oct 21, 2019	Nov 7, 2019	Dec 16, 2019	Mar 30, 2020	May 17, 2020	Jun 12, 2020						
#10									(Jun 23 d)						
	25.6	25.4	25.0	24.7	23.2	21.5	21.5	24.8							
	-	-	Feb 28, 2020	Apr 12, 2020	May 24, 2020	Jun 12, 2020	Jul 3, 2020	Jul 30, 2020	Aug 24, 2020	Sep 23, 2020					
#11										(Nov 5 d)					
	-	-	21.5	21.9	25.1	25.1	25.2	25.8	26.0						
	-	-	Feb 28, 2020	Apr 12, 2020	May 24, 2020	Jun 15, 2020	Jul 6, 2020	Aug 3, 2020							
#12								(Aug 20 d)							
	-	-	21.5	21.9	25.1	25.1	25.2								

#13	-<br>-	-<br>-	Feb 28, 2020<br>21.5	Apr 12, 2020<br>21.9	May 24, 2020<br>25.1	Jun 16, 2020<br>25.1	Jul 8, 2020<br>(Jul 20 d)			
#14	-<br>-	-<br>-	Feb 28, 2020<br>21.5	Apr 12, 2020<br>21.9	May 24, 2020<br>25.2	Jun 10, 2020<br>25.1	Jul 2, 2020<br>25.2	Jul 29, 2020<br>26.0	Sep 4, 2020<br>25.2	Oct 30, 2020<br>(Mar 31, 2021 d)
#15	-<br>-	-<br>-	Feb 28, 2020<br>21.5	Apr 12, 2020<br>21.9	May 24, 2020<br>25.1	Jun 15, 2020<br>25.1	Jul 6, 2020<br>25.2	Aug 3, 2020<br>26.1	Sep 8, 2020<br>(Oct 1 d)	
#16	-<br>-	-<br>-	Feb 28, 2020<br>21.5	Apr 12, 2020<br>21.9	May 24, 2020<br>25.1	Jun 15, 2020<br>25.1	Jul 6, 2020<br>25.2	Aug 3, 2020<br>(Oct 23 d)		
#17	Jun 26, 2020<br>25.1	Jul 1, 2020<br>25.1	Jul 13, 2020<br>25.3	Aug 6, 2020<br>26.2	Aug 30, 2020<br>25.9	Sep 22, 2020<br>a/d				
#18	Jun 26, 2020<br>25.1	Jul 1, 2020<br>25.1	Jul 13, 2020<br>a/d							
#19	Jun 26, 2020<br>25.1	Jul 1, 2020<br>25.1	Jul 15, 2020<br>25.4	Aug 10, 2020<br>26.3	Aug 31, 2020<br>26.0	Sep 17, 2020<br>25.3	Oct 12, 2020<br>24.4	Nov 11, 2020<br>(Jan 26, 2021 d)		
#20	Jun 26, 2020<br>25.1	Jul 1, 2020<br>25.1	Jul 15, 2020<br>25.5	Aug 11, 2020<br>26.2	Aug 31, 2020<br>25.9	Sep 22, 2020<br>25.2	Oct 13, 2020<br>24.3	Nov 14, 2020<br>-	ukn<br>-	Mar 29, 2021<br>(Apr 16 d)
#21	Jun 26, 2020<br>-	ukn<br>-	Jul 16, 2020<br>25.5	Aug 11, 2020<br>26.2	Aug 31, 2020<br>25.9	Sep 22, 2020<br>25.2	Oct 18, 2020<br>24.2	Nov 20, 2020<br>21.3	Feb 15, 2021<br>(Mar 5 d)	
#22	Jun 28, 2020<br>25.2	Jul 2, 2020<br>25.1	Jul 16, 2020<br>25.5	Aug 12, 2020<br>26.2	Aug 31, 2020<br>25.9	Sep 22, 2020<br>25.3	Oct 12, 2020	Nov 9, 2020<br>(Jan 13, 2021 d)		
#23	Jun 28, 2020<br>25.2	Jul 2, 2020<br>25.1	Jul 16, 2020<br>25.5	Aug 12, 2020<br>26.2	Aug 31, 2020<br>25.9	Sep 22, 2020<br>(Oct 1 d)				
#24	Jun 28, 2020<br>-	ukn<br>-	Jul 16, 2020<br>(Aug 12 d)							
#25	Jun 28, 2020<br>-	ukn<br>-	Jul 17, 2020<br>(Aug d)							

#26	Jul 10, 2020<br>25.0	Jul 15, 2020<br>h		
#27	Jul 10, 2020<br>25.0	Jul 15, 2020<br>h		
#28	Jul 10, 2020<br>25.0	Jul 15, 2020<br>h		
#29	Jul 10, 2020<br>25.0	Jul 15, 2020<br>h		
#30	Jul 10, 2020<br>25.0	Jul 15, 2020<br>h		
#31	Jul 12, 2020<br>24.9	Jul 16, 2020<br>h		
#32	Jul 12, 2020<br>24.9	Jul 16, 2020<br>h		
#33	Jul 12, 2020<br>24.9	Jul 16, 2020<br>h		
#34	Jul 12, 2020<br>25.0	Jul 17, 2020<br>h		
#35	-<br>-	Jul 15, 2020<br>25.3	Jul 29, 2020<br>25.8	Aug 25, 2020<br>h
#36	-<br>-	Jul 15, 2020<br>25.3	Jul 30, 2020<br>25.9	Aug 25, 2020<br>h
#37	-<br>-	ukn<br>-	Jul 30, 2020<br>25.9	Aug 26, 2020<br>h
#38	-<br>-	ukn<br>-	Jul 30, 2020<br>26.0	Aug 31, 2020<br>h
#39	-<br>-	ukn<br>-	Jul 30, 2020<br>(Aug 22 d)	

	-	ukn	Jul 31, 2020	Aug 25, 2020
#40				h
	-	-	25.9	

d death that was considered natural.
a/*d* accidental death by mishandling; not counted as natural death.
unk unknown.
h observation halted.
