# Supplementary material for: Development and Reproduction of a Japanese Strain of Ctenolepisma calvum (Ritter, 1910) at Room Temperature
Source: Insects. 2023 Jun 16;14(6):563. doi: 10.3390/insects14060563 (PMC10299600; doi:10.3390/insects14060563)
Supplement: Supplementary file 1 [file insects-14-00563-s001.zip › Table S3-5_230510.pdf]

Table S3. Average instar durations.

**Average temperatures above 22.0 °C**														
**Instar number**	**1**	**2**	**3**	**4**	**5**	**6**	**7**	**8**	**9**	**10**	**11**	**12**	**13**	**14**
Sample size *n*	18	11	15	16	22	17	13	8	2	1	4	2	1	0
Average duration (days)	4.7	13.2	26.1	25.2	23.3	22.6	27.6	33	43	31	40	58	76	-
Standard error	0.1	0.4	0.9	3.0	1.3	0.9	0.9	2	-	-	3	-	-	-
**Average temperatures 22.0 °C or below**														
**Instar number**	**1**	**2**	**3**	**4**	**5**	**6**	**7**	**8**	**9**	**10**	**11**	**12**	**13**	**14**
Sample size *n*	0	0	6	6	0	2	5	5	7	3	0	0	0	1
Average duration (days)	-	-	44	42	-	93	53	68	79	71	-	-	-	151
Standard error	-	-	0	0	-	-	4	9	10	2	-	-	-	-

Table S4. Head width (HW) and growth ratio in each instar.

**Instar number**	**1**	**2**	**3**	**4**	**5**	**6**	**7**	**8**	**9**	**10**	**11**	**12**	**13**	**14**	**15**
Sample size *n*	20	18	22	22	22	22	19	18	14	10	4	4	2	1	1
Average HW (mm)	0.360	0.398	0.442	0.495	0.560	0.641	0.724	0.803	0.862	0.929	0.994	1.057	1.125	1.147	1.155
Standard error	0.002	0.002	0.002	0.003	0.005	0.006	0.008	0.009	0.010	0.010	0.011	0.014	-	-	-
Growth ratio	-	1.10	1.11	1.12	1.13	1.14	1.13	1.11	1.07	1.08	1.07	1.06	1.07*	1.02**	1.01**

* Ratio of average HW values of only two individuals, #3 and #4, that developed to the 13th instar.
** Ratio of HW values of only one individual, #3, that developed to the 14th and 15th instar.

Table S5. Instar-specific survival rates at the 3rd and later instars.

**Instar number**	**3**	**4**	**5**	**6**	**7**	**8**	**9**	**10**	**11**	**12**	**13**	**14**	**15**
Sample size *n*	24	22	22	21	19	18	14	10	4	4	2	1	1
Survival rate	0.88	1.0	1.0	0.90	0.95	0.78	0.71	0.40	1	0.5	0.5	1	0
